# Health and Disease

**Published:** 1859-03

**Authors:** 


"*Health and Disease.*"

---

### "HEALTH AND DISEASE."

This is the title of our new book, composed by urgent and repeated request. After it was prepared, the manuscript was submitted to some of the oldest and most respectable publishers of New York, but they all, with one exception, began to make excuse; whereupon we became greatly encouraged, and on making another effort, sold the whole edition to Mr. Price, for about three times as much as the ordinary rates would have yielded us, and thus far he has had no occasion to repent of his bold and liberal offer, for his sagacity has been already proven in the encouraging sale and high appreciation of the book, by some of the best minds and competent judges in the city. In refusing to touch our bantling, the publishers, like all sensible men, were very polite, very courteous—in fact, quite complimentary of our genius, ability, and all that, but firmly held off, notwithstanding.

The book is useful and truthful, chaste in idea, sound in doctrine "Allopathic;" appealing constantly to the observation and common sense of the reader. It never advises a dose of medicine, does not recommend expensive appliances, does not ride on the hobby of starving a man to death—of denying coffee and tea and roast beef. It wars relentlessly against the patronage of uneducated physicians, of quackery and of patent medicines in all their forms; it discourages self-medication, shows its tendencies and its dangers, and plainly inculcates the practice of never taking an atom of medicine, except by the advice of a respectable physician ; but not to seek that advice until the natural agencies of air, exercise, temperance, cleanliness, rest, and warmth, have been fully employed, according to the directions given.

Furthermore, " Health and Disease" contains advice which every human being needs ; advice which needs to be put in practice every day of his existence ; advice, the lack of which is resulting daily in the early loss of health and premature death of multitudes ; advice which has, perhaps, never yet been given in any printed book for popular use ; advice which tens of thousands now living would give a large share of all they possess, had they known and practised it before their misfortunes came upon them.

Why, then, would not the long-headed publishers of New York take hold of this book, and risk on it three hundred and seventy-five dollars on the first thousand copies, retailing at one dollar? And why was it that in the face of these hard facts we did not throw the manuscript in the fire, and vote that " every publisher in the universal world was a fool, and had no sense?"

In the first place, we knew that publishers, like politicians, never work for the people ; they work for themselves ; the only question which they ever busy themselves in answering is, " Will it pay?" But to answer that with any certainty, their guide must be their experience ; and long since it became an axiom in " the trade," that books, whose aim was the solid benefit and permanent advantage of the people, were uniformly published at a loss. Now this is not the fault of the publishers, but of the people themselves ; and as publishers, like other people, have families to support, food to eat, houses to live in, children to educate, and baby shoes to buy, the laws of self-preservation, long-headedness, and rhinocerocity of hide and conscience, dictate a falling in with the tide of public demand, and not of public need. Flashy novels pay—so does the yellow-covered literature. Periodicals containing splendid fashion plates of monstrosities, only worn by pimps and courtezans, and people "from the country"—that is, outside of cities—these do well; and quite as profitable are sensation weeklies, with blood and murder stories of land and sea, illustrated "in wood" by pictures of indecency, profanity, and blood. So do papers well pay which spread abroad by the million impossible stories of the "spirit world," the falsities of "water cure," and the atheisms of phrenology; but when it comes to books which treat of the "uses and abuses of air," of a rational temperance, which shows how, in a timely manner, to avoid disease and to cure it when present by prudence, patience, and a wise self-denial, a conservative, let-alone-ism, such books remain on the shelf of a humane publisher to gather dust and mold, and finally to be eaten of worms.

But with all these things before his eyes, Mr. Price had no fear that the book would not sell. He knew that though all the fools were not dead yet, there were sensible people at magnificent distances, and that these would derive practical advantage from reading the book, and, doing so, would not fail to recommend it to others. Besides, we gathered courage from the fact that while it was in course of publication in friend Gray's mammoth establishment in Jacob street, it made converts among every class of operatives, whose duties required a perusal of its pages in composing, setting up, correcting, &c. We judged from this that there was persuasive eloquence in it; that those of very moderate education could easily understand it, and that their personal experiences compelled a conviction of its truth. This was enough; for we wanted to reach the capacity of the masses, and we have succeeded.

It is a book not for the day and hour, but for the age. It will be as true and practical and necessary in the year nineteen hundred as now, for it advises temperance, without starvation; enjoyment, without satiety; mirth, without tom-foolery; exercise, without exhaustion; and piety, without pretence. It aims to show that constitutions impaired and broken in early life may, by means of natural agencies, be built up again, to last to a green old age, and that by these same means the ordinary ailments of dyspeptia, neuralgia, constipation, susceptibility to colds, chilliness, &c., can be safely, efficiently, and permanently cured.

